# The Use of Chitosan to Enhance Photodynamic Inactivation against *Candida albicans* and Its Drug-Resistant Clinical Isolates

**DOI:** 10.3390/ijms14047445

**Published:** 2013-04-03

**Authors:** Hsiung-Fei Chien, Chueh-Pin Chen, Yee-Chun Chen, Po-Han Chang, Tsuimin Tsai, Chin-Tin Chen

**Affiliations:** 1 Department of Surgery, National Taiwan University College of Medicine, Taipei 100, Taiwan; E-Mail: hfchien@ntu.edu.tw; 2 Department of Biochemical Science and Technology, National Taiwan University, Taipei 106, Taiwan; E-Mails: d00b22004@ntu.edu.tw (C.-P.C.); todd0928@hotmail.com (P.-H.C.); 3 Department of Internal Medicine, National Taiwan University College of Medicine, Taipei 100, Taiwan; E-Mail: yeechunchen@gmail.com; 4 Graduate Institute of Biomedical Materials and Tissue Engineering, College of Oral Medicine, Taipei Medical University, Taipei 110, Taiwan; E-Mail: tmtsai00@tmu.edu.tw

**Keywords:** chitosan, *Candida*, antimicrobial, photodynamic inactivation

## Abstract

Drug-resistant *Candida* infection is a major health concern among immunocompromised patients. Antimicrobial photodynamic inactivation (PDI) was introduced as an alternative treatment for local infections. Although *Candida* (*C.*) has demonstrated susceptibility to PDI, high doses of photosensitizer (PS) and light energy are required, which may be harmful to eukaryotic human cells. This study explores the capacity of chitosan, a polycationic biopolymer, to increase the efficacy of PDI against *C. albicans*, as well as fluconazole-resistant clinical isolates in planktonic or biofilm states. Chitosan was shown to effectively augment the effect of PDI mediated by toluidine blue O (TBO) against *C. albicans* that were incubated with chitosan for 30 min following PDI. Chitosan at concentrations as low as 0.25% eradicated *C. albicans*; however, without PDI treatment, chitosan alone did not demonstrate significant antimicrobial activity within the 30 min of incubation. These results suggest that chitosan only augmented the fungicidal effect after the cells had been damaged by PDI. Increasing the dosage of chitosan or prolonging the incubation time allowed a reduction in the PDI condition required to completely eradicate *C. albicans*. These results clearly indicate that combining chitosan with PDI is a promising antimicrobial approach to treat infectious diseases.

## 1. Introduction

Chitosan is a natural linear polycationic biopolymer comprising N-acetyl-D-glucosamine and β-1,4-linked D-glucosamine. In dilute acid solutions, the positive charge of chitosan interferes with the negatively charged residue of macromolecules on the surface of cells [1], presumably by competing with Ca^2+^ for electronegative sites on the membrane without conferring dimensional stability or rendering the membrane leaky [1]. In general, the bacteriostatic/bactericidal activity of chitosan is related to the protonated positive charge number of chitosan and the number of negative charges on the surface of the microbe [1,2]. The antimicrobial activity of chitosan was found against a wide variety of fungi, yeasts and bacteria [3]. Because of its safety and excellent biocompatibility [4], chitosan has been used in food preservation [3], medical and pharmaceutical applications for wound healing [5], drug delivery [6] and tissue engineering [7].

*Candida* is an opportunistic pathogen in humans. In recent years, fungal infection has become a leading cause of nosocomial infections [8,9], responsible for a range of diseases from superficial mucosal to systemic disorders. *Candida* infection is also encountered in immunocompromised patients, such as those with HIV [10] and severe burns [11]. In addition, implants and prostheses often harbor biofilms of *C. albicans*. Although antibiotics against fungal infection are available, resistant strains are frequently reported. Therefore, there is a compelling need for alternative antimicrobials effective in the treatment of multi-drug resistant fungal infections. Recently, the use of chitosan to control postharvest and phytopathogenic fungal disease has attracted much attention, due to imminent problems associated with chemical agents. The fungicidal activity of chitosan varies considerably according to type of chitosan, the host targeted and the environment in which it is applied [3]. N-carboxybutyl chitosan was shown to disturb the membrane functions of *C. albicans*[1,2]. In addition, plain 2% chitosan gel demonstrated the least inhibition of *C. albicans*[12]. However, the antimicrobial studies of chitosan against pathogenic *Candidas* are still limited.

Antimicrobial photodynamic inactivation (PDI) has been developed as an alternative therapeutic tool against bacterial infection, garnering considerable interest in the management of drug-resistant bacterial strains [13,14]. PDI as a bactericide employs a combination of nontoxic photosensitizer (PS) and visible light to generate cytotoxic species. Following light irradiation, the activated PS reacts with molecules in its direct environment, either through electron transfer producing free radicals (type I reaction) or through energy transfer, generating highly reactive singlet oxygen in the presence of oxygen (type II reaction). Unlike other therapies, PDI provides the advantage of dual selectivity; the PS is targeted to the infected area, and the light can be accurately directed to the affected tissue [13]. Furthermore, PDI causes direct damage to the cell wall and membranes, because of the direct binding of PS to these structures. Thus, targeted cells have little chance of developing resistance or increasing metabolic detoxification or export of the drug [15]. Evidence indicates that repeated PDI does not induce resistance in the bacteria against PDI treatment [16,17].

Eukaryotic species, such as *C. albicans*, are less susceptible to killing with PDI than prokaryotic bacteria [18]. C*andida* species are approximately 25~50-times larger than bacterial test species and, therefore, contain a larger number of targets per cell [18]. This may explain why *C. albicans* was shown to be susceptible to toluidine blue O (TBO) and methylene blue (MB) mediated PDI at higher doses of photosensitizers [19]. It has been demonstrated that PDI with MB or TBO, under conditions conducive to the effective killing of typical skin microbes, causes neither cytotoxicity [18,20,21] nor DNA damage to keratinocytes *in vitro*[22]. However, both humans and *Candida* are eukaryotic, and higher doses of photosensitizers or light irradiation might still be harmful to human cells.

In a previous study, we showed that chitosan can potentiate the efficacy of PDI against both Gram**-**(+) and Gram**-**(−) prokaryotic bacteria in planktonic cells and biofilms [23]. In the present study, the utility of chitosan in PDI against eukaryotic *C. albicans* was evaluated. Chitosan was shown to potentiate the efficacy of PDI in planktonic cells and biofilms of *C. albicans*. Following the administration of PDI, the addition of chitosan greatly augmented the killing of *C. albicans* and drug-resistant clinical isolates. Because the safety of chitosan as a biomaterial is well known, the combination of chitosan and PDI for the treatment of fungal infections shows considerable promise.

## 2. Results and Discussion

### 2.1. TBO Binding Assay and Survival Fraction as Related to Incubation Time and Dose

**Figure 1:**
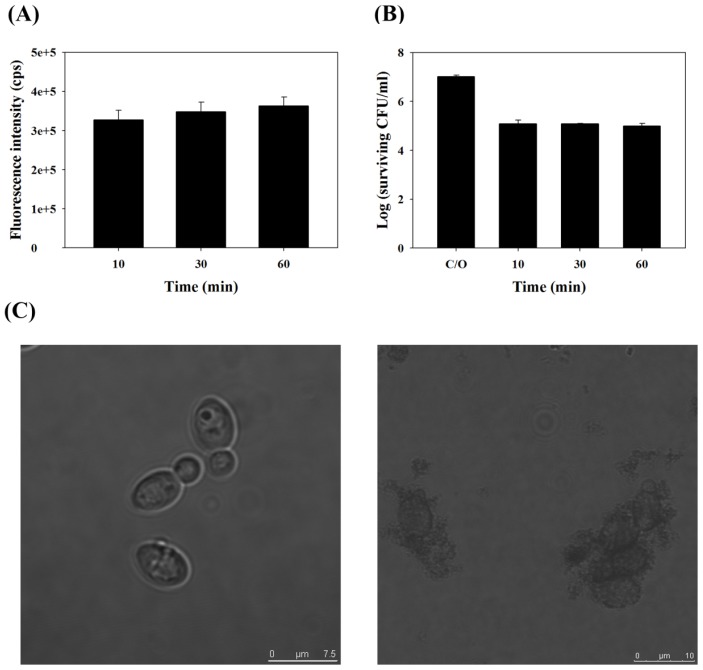
Toluidine blue O (TBO) binding (**A**) and survival fraction (**B**) of *C. albicans* were incubated with 0.2 mM TBO for different time and then measured with a spectrophotometer for the TBO binding (**A**) or subjected to 50 J/cm^2^ of the red light illumination. Each value is the mean from three independent experiments ± standard deviation. Neither fluorescence nor photodynamic inactivation (PDI) significantly increased as incubation time increased; (**C**) Morphology of *C. albicans* before (left panel) and after (right panel) PDI, as observed by confocal microscope. *C. albicans* was incubated with TBO (0.2 mM, 30 min), followed by red light illumination (50 J/cm^2^).

To examine the binding of TBO to *C. albicans* and the survival fraction as it relates to incubation time, we measured the intensity of fluorescence after incubating 1 × 10^7^ CFU/mL *C. albicans* with 0.2 mM TBO for 10, 30 or 60 min. As shown in Figure 1A, the binding of TBO to *C. albicans* was significant and remained steady after incubation for 10 min. Following light irradiation (50 J/cm^2^), the surviving fraction decreased after 10 min of incubation with TBO and remained steady at 30 and 60 min (Figure 1B). Neither PS binding nor PDI increased significantly with an increase in incubation time, indicating that TBO binding and its subsequent PDI effect occurred very quickly, if not instantaneously. For the convenience of the following experiments, 30 min was selected as the standard incubation time.

Analysis of the morphology of *C. albicans* through a confocal microscope was also carried out before and after PDI. Prior to illumination, the cell wall of *C. albicans* was intact and regular in shape (left panel, Figure 1C). However, the cell walls were fragmented and irregular in shape after PDI (right panel, Figure 1C). The cell walls of a number of *C. albicans* appeared swollen or disintegrated.

### 2.2. Chitosan Augments TBO or Ce6 Mediated PDI against Planktonic *C. albicans*

**Figure 2:**
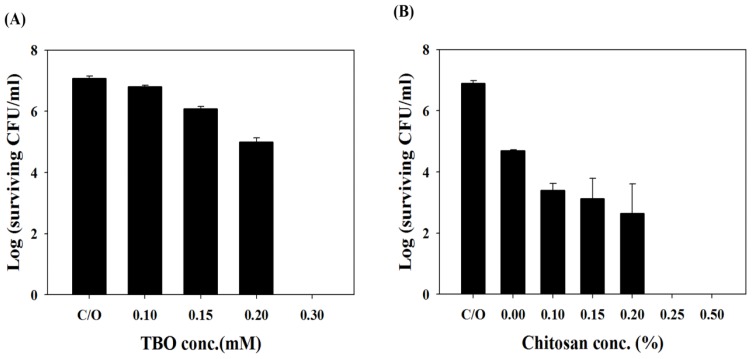
(**A**) Cell survival fraction of planktonic *C. albicans* after being incubated with different concentrations of TBO for 30 min and subjected to 50 J/cm^2^ of the red light illumination; (**B**) Cell survival fraction of planktonic *C. albicans* after PDI (0.2 mM TBO and 50 J/cm^2^) followed by treatment with various concentrations of chitosan for 30 min and then plate counted. Each value is the mean from three independent experiments ± SD.

The impact of TBO concentration on PDI against *C. albicans* was found to be dose-dependent (Figure 2A). PDI is only capable of causing approximately 1- and 2-log_10_ reductions in viable cell count using 0.15 mM and 0.2 mM TBO, respectively. PDI with TBO at 0.3 mM completely eradicated *C. albicans*.

**Figure 3:**
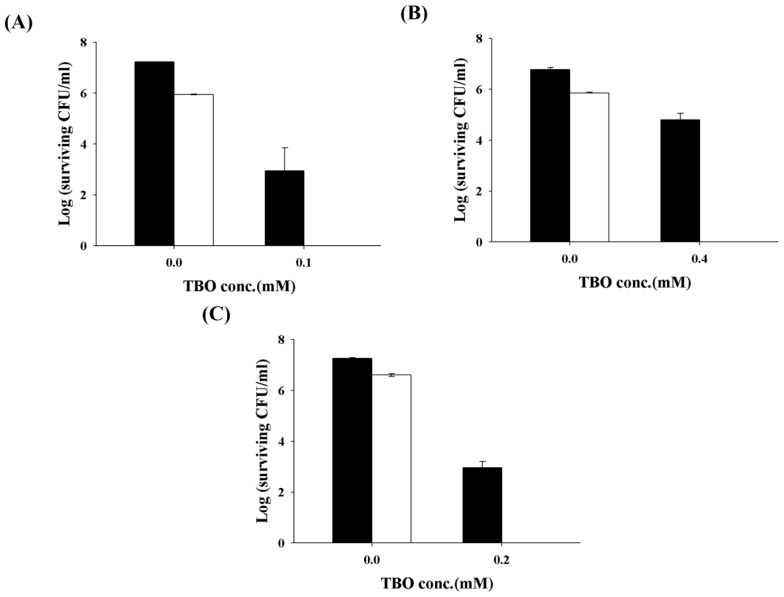
Effect of chitosan on TBO-mediated PDI against fluconazole-resistant *C. albicans* strain 2008 no30 (**A**), 2008 no22 (**B**) and 2008 no19 (**C**). TBO at 0.1 mM, 0.4 mM and 0.2 mM were incubated with strain 2008 no30, no22 and no19 for 30 min, respectively. After light illumination, cells were incubated with 0.25% chitosan for 30 min and then plate counted. Closed column: PDI only. Open column: PDI plus chitosan. Each value is the mean from three independent experiments ± SD.

Antimicrobial PDI has provided new hope for controlling microbial infections. The main advantages of PDI are the ability to eradicate bacteria almost instantly, while avoiding damage to adjacent host tissue. A number of PSs have demonstrated the efficacy of PDI against microbial pathogens; however, many commonly used PSs, capable of significant phototoxicity against Gram**-**(+) bacteria, are ineffective against Gram**-**(−) bacteria, due to the properties of their outer membrane. In a previous study, we showed that encapsulating PSs in liposomes or micelles can improve their photochemical efficiency and subsequently enhance the efficacy of PDI in killing Gram-(+) bacteria [24]. In a recent study [23], we further determined that one of the most commonly studied biomaterials, chitosan, can potentiate the efficacy of PDI against Gram**-**(+) or Gram**-**(−) bacteria. As the cell wall compositions of *C. albicans* are quite different from bacteria, we wonder whether chitosan can exert the augmented effect in PDI against *Candida*. Therefore, various concentrations of chitosan were added following PDI with 0.2 mM TBO. As shown in Figure 2B, without chitosan incubation, PDI only devitalized *C. albicans* on a scale of 2-log_10_. However, the fungicidal effect was augmented with chitosan in a dose-dependent manner and resulted in complete killing after incubation for 30 min in 0.25% chitosan following PDI. The augmented effects of chitosan in PDI were also found in three clinical isolates of fluconazole-resistant *C. albicans* (Figure 3). The potentiated effects of chitosan in PDI are valuable, particularly when the photosensitizer is expensive or toxic to normal human tissue in clinical practice.

### 2.3. Chitosan Augments TBO Mediated PDI against the Biofilm of *C. albicans*

**Figure 4:**
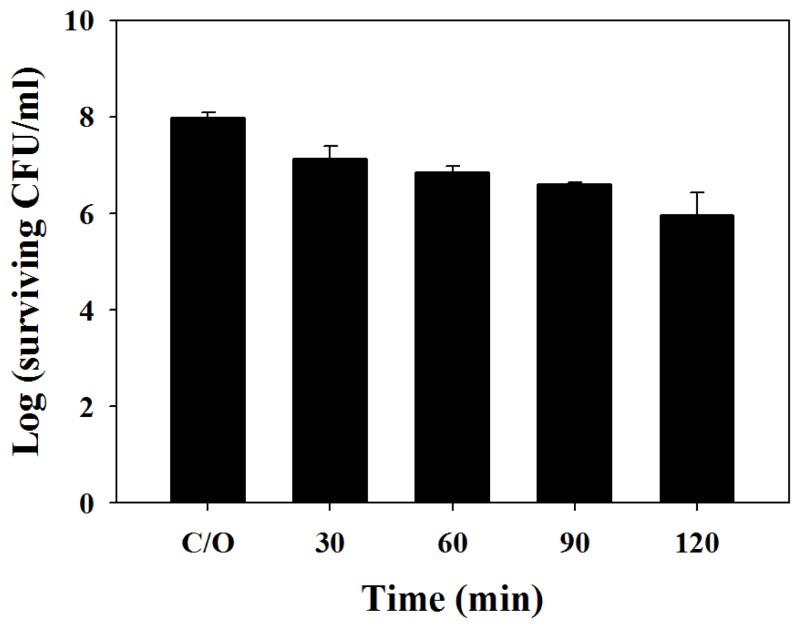
Cell survival fraction of *C. albicans* biofilm after TBO-mediated PDI. Biofilm cells were treated with 20 mM TBO for different periods of time, followed by light exposure at 100 J/cm^2^. Each value is the mean from three independent experiments ± SD.

**Figure 5:**
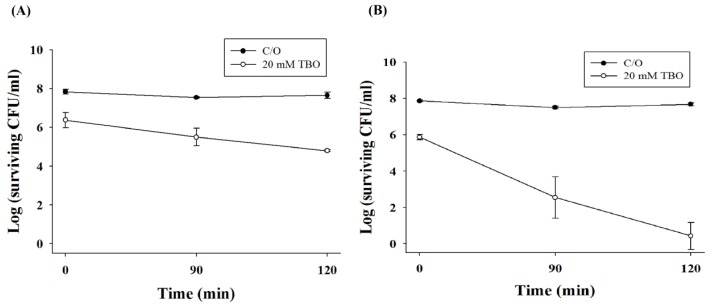
Effect of TBO-mediated PDI followed by chitosan treatment on biofilm of *C. albicans.* Biofilm cells were treated with 20 mM TBO for 1 h (**A**) or 2 h (**B**), followed by light exposure at 100 J/cm^2^. After PDI, biofilm cells were further treated with 0.5% chitosan for 90 and 120 min and then subjected to a plate count. Each point is the mean from three independent experiments ± SD.

We further examined the potentiating effect of chitosan in PDI against the biofilms of *C. albicans*. Under a light energy dose of 100 J/cm^2^, 1- and 2-log_10_ reductions in viable counts were found in biofilm cells incubated with 20 mM TBO for 1 h or 2 h, respectively (Figure 4). However, the killing of biofilm cells of *C. albicans* increased following incubation with chitosan (0.5%, *w*/*v*) for 90 or 120 min (Figure 5). Complete killing of *C. albicans* in the biofilm was observed after the incubation of chitosan for 2 h following PDI with a TBO incubation time of 2 h (Figure 5B), suggesting that the augmentation of chitosan in PDI against biofilm cells is related to the level of damage. It has been reported that the biofilm of *C. albicans* is resistant to most antifungal drugs [25,26]. This explains why the required TBO concentration in the biofilm study is 100-times higher than that of planktonic cells. Taken together, these results clearly indicate that chitosan can potentiate the efficacy of PDI against planktonic, as well as biofilm cells.

**Figure 6:**
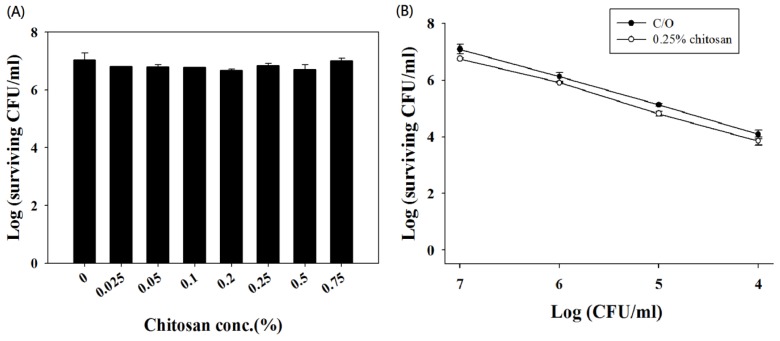
Chitosan alone did not demonstrate antimicrobial activity. (**A**) Chitosan of various concentrations did not influence the viability of *C. albicans* cells. Cells at a concentration of 1 × 10^7^ CFU/mL were not affected after incubation with chitosan as high as 0.75% for 30 min; (**B**) Chitosan of 0.25% did not influence the viability of *C. albicans* at various cell densities. Data are the means of three independent experiments and the bars are the SDs.

Although the antimicrobial activity of chitosan alone has been demonstrated, a complete lack of activity against microbes was observed after 18–48 h of incubation [3]. However, without PDI treatment, we found that various concentrations of chitosan alone did not demonstrate significant toxicity against *C. albicans* after 30-min incubation (Figure 6A). It has been reported that the concentration of chitosan required to exert antimicrobial activity varies according to the species and concentration of the microorganism. In this regard, it is possible that the potentiating effect of chitosan might be due to the reduced number of surviving *C. albicans* cells after PDI. To examine this further, various cell densities of *C. albicans* were incubated with 0.25% (*w*/*v*) chitosan for 30 min. However, no antimicrobial activity against *C. albicans* was observed (Figure 6B). These results indicate that the potentiating effect of chitosan worked after the *C. albicans* cells were damaged by PDI and was not associated with a decreased number of surviving cells.

Many studies have reported on the mechanisms underlying the antimicrobial activity of chitosan [27]. Park *et al.* reported that low molecular weight water-soluble (LMWS) chitosan exhibits antifungal activity, but no cytotoxicity against mammalian cells [28]. Using confocal microscopy, they showed that LMWS-chitosan is located in the plasma membrane and demonstrates membrane disrupting activity. In our analysis, using an electron microscope, we also observed that chitosan binds to the cell wall with or without PDI (data not shown). However, we observed no cell killing without PDI damage to the cells.

For clinical applications, an ideal antimicrobial PDI should exhibit extensive killing of the pathogen population with minimal damage to host tissues in the area of infection. Although PSs are capable of exerting phototoxic effects on microbes, damage to neighboring human cells is still possible if the PS dose is too high. It would be ideal if a lower safe dose of PS could function efficiently as a microbial killing agent. The potentiating effect of chitosan in TBO-mediated PDI against *C. albicans* either in planktonic cells or biofilms was demonstrated in this study, indicating that when PDI is combined with chitosan, a lower dose of PS and light may function effectively as a microbial killing agent. Chitosan is abundant in nature, and most studies have shown it to be non-toxic to mammalian cells. The combined use of chitosan with PDI offers a new direction for the eradication of all microbes, while preventing mutagenesis among survivors.

## 3. Experimental Section

### 3.1. Materials

Low-molecular-weight chitosan (MW ~20 kDa) with a degree of deacetylation (DDA) of ~90% was purchased from Shin Era Technology (Taipei, Taiwan). Toluidine blue O (TBO) and all other chemicals were obtained from Sigma-Aldrich (St. Louis, MO, USA).

### 3.2. Candida Strains and Growth Conditions

Three strains of *C. albicans*, namely, a wild-type strain SC5314 (ATCC MYA-2876D, kindly provided by L.Z. Den, Department of Medical Technology, National Taiwan University, Taipei, Taiwan) and three fluconazole-resistant clinical strains (2008 no. 19, 22 and 30) from the infection control lab at the National Taiwan University Hospital, Taipei, Taiwan, were used. *C. albicans* strains were grown aerobically overnight (12 h) in 50 mL yeast peptone dextrose (YPD) broth at 37 °C. Cells were then harvested following centrifugation at 5000× *g* for 5 min, washed three times with phosphate-buffered saline (PBS; pH 7.4) and suspended in PBS to produce a cell suspension containing 10^7^ CFU/mL.

### 3.3. Biofilm Preparation

Biofilms of *C. albicans* were developed according to a published protocol [29]. In brief, washed *C. albicans* cells were resuspended in Roswell Park Memorial Institute (RPMI) 1640 medium, supplemented with 1% glucose and adjusted to a cell density of approximately 1 × 10^7^ cells/mL. Then, 100 μL of cell suspension was pipetted into each well of a 96-well polystyrene flat plate and shaken at 75 rpm for 1.5 h at 37 °C to permit the cells to adhere to the surface of the wells. Cell suspensions were then aspirated and each well was washed with 100 μL PBS to remove loosely adherent cells. A 200 μL RPMI 1640 medium supplemented with 1% glucose was pipetted into the washed wells. The plates were then incubated at 37 °C and shaken at 75 rpm for 48 h.

### 3.4. TBO Incubation

A cell suspension of *C. albicans* containing approximately 1 × 10^7^ CFU/mL was mixed with equal volumes of TBO of various concentrations, cultured at 25 °C for 30 min in the dark and then centrifuged at 12,000× *g* for 1 min. Cells were then suspended in PBS. To perform a binding assay, the sample was lysed with lysis buffer (0.1 N NaOH-1% SDS) and held in the dark for 24 h at room temperature before being measured using a spectrophotometer (FluoroMax^®^-4, Horiba Jobin Yvon, NJ, USA) at an excitation wavelength of 624 nm. Fluorescence emission was measured at wavelengths ranging from 580 to 700 nm.

### 3.5. PDI in Planktonic Cells of *C. albicans*

In a typical experiment, 0.1 mL of *C. albicans* cell suspension containing approximately 10^7^ CFU/mL was transferred into a well. Then, 0.1 mL of PBS solution (pH 7.4) containing TBO was added to the solution. Samples were incubated in the dark for 30 min at 25 °C and shaken at 100 rpm, unless otherwise specified. Samples were then centrifuged (12,000 × *g*, 1 min), washed once with PBS and resuspended using 200 uL PBS. The home-made light source used for TBO irradiation consisted of a high-power LED array with the wavelength centered at 630 ± 5 nm, delivered at an irradiance of 30 mW/cm^2^ and 50 J/cm^2^. Irradiated and non-irradiated samples were serially diluted 10-fold with PBS, and the colonies that formed on YPD agar plates after 18 h of incubation at 37 °C were counted. Analysis of morphology using a confocal microscope was carried out after *Candida* was first co-incubated with TBO (0.2 mM) and then treated with PDI using 50 J/cm^2^ (630 ± 5 nm, 30 mW) of red light.

### 3.6. TBO Mediated PDI in Biofilm Cells

Disks with biofilms were placed in a sterile 48-well microtiter plate and treated with 0, 20 and 40 mM of TBO in the dark for 30 min. Disks were then moved to a new plate containing PBS and irradiated using an LED array (630 ± 5mm, 30 mW) at 50 J/cm^2^. Following irradiation, the disk with biofilms were then placed into test tubes containing 10 mL sterile PBS and vigorously vortexed to remove the biofilm from the disks. The resulting microbial suspensions were suitably diluted and plated on tryptic soy agar. Colonies that formed after 18 h of incubation at 37 °C were counted.

### 3.7. Effect of Chitosan on PDI

A stock solution of chitosan (1% *w*/*v*) was prepared in 1% acetic acid and used within 1 month. Various concentrations of chitosan were prepared by taking aliquots from the chitosan stock and diluting them with culture medium. Chitosan was added to the cells after performing PDI. Following incubation for various durations, cells were washed out using fresh culture medium. In the presence or absence of chitosan, irradiated and non-irradiated *Candida* cells were serially diluted 10-fold with PBS, and the colonies that formed after 18 h of incubation at 37 °C were counted.

### 3.8. *C. albicans* Cell Survival Assay

Colony-forming units of *Candida* suspensions were counted using the following protocol: aliquots (10 μL) of appropriate dilutions (from 10^−1^ to 10^−5^) were plated on YPD agar plates and incubated at 37 °C in the dark for 18 h. The surviving fraction was calculated as N_PDI_/N_0_, where N_PDI_ is the cell count (CFU/mL) after antimicrobial photodynamic therapy and N_0_ is the cell count (CFU/mL) in the initial sample. The toxicity of substrates in the dark, defined as the intrinsic toxicity of the compounds in the absence of light, was monitored by evaluating the surviving fraction of non-illuminated *C. albicans* samples and calculated as N_DARK_/N_0_, where N_DARK_ is the cell count (CFU/mL) of the non-illuminated samples. All results were expressed as the mean ± SD. Differences between two means were assessed for significance using the two-tailed Student’s *t*-test, and *p* < 0.05 was considered significant.

## 4. Conclusions

The combination of PDI and chitosan was shown to potentiate the PDI efficacy in planktonic cells and biofilms of *C. albicans*. Due to the characteristics of biocompatibility and saltiness, chitosan is quite promising in augmenting the PDI efficacy for eradicating *C. albicans* infection.
